# Genetic characterization of a global collection of common buckwheat to harness genetic resources for agricultural diversity

**DOI:** 10.3389/fpls.2026.1902211

**Published:** 2026-09-16

**Authors:** Fabian Hess, Lukas Kronenberg, Steven Yates, Daniel Ariza-Suarez, Michelle M. Nay, Heidi Heuberger, Bianca Büttner, Friedrich J. Zeller, Christian Eigenmann, Achim Walter, Bruno Studer

**Affiliations:** 1 Molecular Plant Breeding, Institute of Agricultural Sciences, ETH Zurich, Zurich, Switzerland; 2 Precision Phenotyping, Institute of Agricultural and Nutritional Science, Martin Luther University Halle-Wittenberg, Halle (Saale), Germany; 3 Fodder Plant Breeding, Division of Plant Breeding, Agroscope, Zurich, Switzerland; 4 Institute for Crop Science and Plant Breeding, Bavarian State Research Center for Agriculture, LfL, Freising, Germany; 5 Plant Breeding, Centre for Food and Life Sciences Weihenstephan, Technical University of Munich, Freising, Germany; 6 Genetic Resources, Production Security and Animal Feed Unit, Federal Office for Agriculture FOAG, Bern, Switzerland; 7 Crop Science, Institute of Agricultural Sciences, ETH Zurich, Zurich, Switzerland

**Keywords:** agricultural diversity, common buckwheat (*F. esculentum* Moench), plant genetic resources, Genome-wide allele frequency fingerprints (GWAFFs), neglected and underutilized crops

## Abstract

**Introduction:**

Neglected and underutilized crops have great potential to diversify agroecosystems and human diets but require genetic improvement to make them economically viable alternatives to major crops. Common buckwheat (*Fagopyrum esculentum* Moench) is valued for its excellent nutritional quality, its ecological importance and its benefits on crop rotation and soil health. However, low and highly variable yields, along with uneven seed ripening, have limited the crop’s usage in farming systems, highlighting the need for improved varieties. The availability of genetic resources and detailed genetic characterization are prerequisites for efficient breeding strategies.

**Methods:**

In this study, we elucidate the genetic diversity and phylogeography of a global germplasm collection comprising 170 common buckwheat accessions, including nine self-compatible accessions and eleven accessions with determinate growth habit. The collection was genetically characterized using double digest restriction-site associated DNA sequencing to generate accession-specific genome-wide allele frequency fingerprints (GWAFFs).

**Results:**

Using accurate and repeatable GWAFFs based on 52,182 genome sites, an analysis of genetic admixture revealed seven subclusters within the collection. Our results show that common buckwheat accessions collected in specific regions of the world usually trace back to a single subcluster. Western Europe is an exception, as accessions from several different subcluster have been integrated into local cropping and food systems.

**Discussion:**

These findings illustrate that most breeding programs for common buckwheat rely on only a fraction of the available genetic diversity. Therefore, the introduction of genetic material from different sources could increase the diversity of many local breeding programs. The precise genetic characterization of the collection with GWAFFs opens opportunities to apply modern breeding strategies such as genomics-assisted selection. True-to-type seed of 81 accessions of the collection were deposited in the Swiss National Gene Bank and are publicly available for cultivation and use in research and breeding.

## 1 Introduction

Mechanization of agriculture has not only led to a massive increase in labor productivity and, consequently, reduced poverty (Agarwal, 1981; Pingali, 2007), but has also resulted in a reduction of diversity in agricultural systems and an increased dominance of a few major crops at the expense of so-called neglected and underutilized crops (NUCs) (FAO, 2019; Hunter et al., 2019). This had negative impacts on biodiversity, ecosystem stability and human nutrition (IPBES, 2019). Common buckwheat (*Fagopyrum esculentum* Moench) is an example of a NUC that has a long cultivation history in many European countries and was still widely grown in the 19^th^ century (Aufhammer et al., 1995; Miedaner and Longin, 2017; Strahm et al., 2019). However, it was increasingly replaced by high-yielding cereals and potato (*Solanum tuberosum* L.) at the beginning of the 20^th^ century and is nearly absent from arable lands in Western Europe today (Zeller and Hsam, 2004; Klerk et al., 2015). The reintroduction of NUCs such as common buckwheat is a possible way to reverse the trend of declining agrobiodiversity and increase the resilience of agroecosystems (Li and Siddique, 2018; Shah et al., 2021; Liu et al., 2022; Yang et al., 2024).

Common buckwheat has several characteristics that justify efforts for its reintroduction. Its short growing season of three to four months and positive effects on soil health make common buckwheat a beneficial addition to many crop rotations (Lichtenhahn and Dierauer, 2000; Farooq et al., 2016). It also offers ecological benefits by serving as a melliferous crop and reducing reliance on plant protection products for pest and disease control (Campbell, 1997; Halbrecq et al., 2005; Costanzo, 2019). In human nutrition, common buckwheat is a gluten-free alternative to cereals (Joshi et al., 2019). It also possesses a favorable amino acid composition compared to most cereals and has high concentrations of health-benefiting secondary metabolites (Kreft et al., 2016; Škrabanja and Kreft, 2016; Jha et al., 2024). Besides its nutritional qualities, common buckwheat also holds great cultural value and plays an important part in regional culinary traditions around the world, such as galettes in France, pizzoccheri in the Alps, kasha in Eastern Europe and soba in Japan (Kreft et al., 2007). The journey of common buckwheat from China across the globe reflects the patterns of humanity’s agricultural and migration history (Klerk et al., 2015; Verschuer, 2016; Hunt et al., 2018). However, low and highly variable yields, along with uneven seed ripening, are currently limiting the crop’s usage in farming systems (Jacquemart et al., 2012), threatening this agronomically, ecologically, nutritionally and culturally valuable crop. Improved varieties are needed to reverse these trends and reinvigorate common buckwheat cultivation (Joshi et al., 2019). The availability of genetic resources and detailed genetic characterization are prerequisites for broader cultivation and efficient breeding strategies.

Efforts to accelerate common buckwheat breeding face economic as well as biological challenges. The limited cultivation area of common buckwheat means that investments into breeding are not financially attractive compared to major crops due to limited revenues through royalties (Campbell, 1997; Yabe and Iwata, 2020). On the technical side, the self-incompatibility of common buckwheat means that accessions represent populations of genetically distinct individuals. Therefore, genotyping approaches have to be based on population-level genome-wide allele frequency fingerprints (GWAFFs, Byrne et al., 2013) which poses additional challenges compared to the genetic analysis of inbred lines. The technical feasibility of generating GWAFFs based on double digest restriction associated DNA (ddRAD) sequencing data of pooled samples of common buckwheat has been demonstrated previously (Nay et al., 2020). Self-incompatibility and annuality also represent challenges by limiting the breeding to population breeding approaches, complicating the fixation of desirable alleles in breeding germplasm and slowing down genetic gain (Aii et al., 1998; Yabe and Iwata, 2020). The lack of genetic improvement means that agronomic shortcomings of common buckwheat such as low and unpredictable yields remain unresolved. Therefore, it is crucial that public investments are not only directed towards the collection and conservation of germplasm (Joshi et al., 2021) but focus on making these genetic resources easily available and accessible for interested stakeholders. Emerging areas of research such as gene bank genomics and crop translational genomics aim at supporting this process, speed up the transition from the gene bank into breeding programs and enable the application of genomics-assisted breeding methods (Mascher et al., 2019, 2024). The genetic characterization of genomic resources of NUCs is especially important as fast agronomic improvement is needed to make them competitive, promote their utilization and harness their potential to contribute to more diverse agroecosystems in the future (Salgotra and Chauhan, 2023).

This study aims to increase the currently limited availability and accessibility of genetic resources of common buckwheat and genetically characterize a large collection of 170 common buckwheat accessions of world-wide origin (hereafter referred to as the ETH Zurich/FOAG collection, with FOAG denoting the Federal Office for Agriculture in Switzerland) at the genomic level using ddRAD sequencing. This data is used to explore the collection’s phylogenetic structure, origins and diversity, and analyze genetic differences between subclusters within the collection. The genetic characterization of the collection is an important prerequisite to advance future research and breeding of common buckwheat. Moreover, the collection is accessible to farmers, breeders and scientists, as true-to-type seed were multiplied in isolation tents and deposited in the Swiss National Gene Bank of Plant Genetic Resources for Food and Agriculture (PGRFA).

## 2 Materials and methods

### 2.1 The ETH Zurich/FOAG collection

Over the years, 170 accessions of common buckwheat from many different sources have contributed to the ETH Zurich/FOAG collection. A collection of 101 accessions assembled in sampling expeditions during 1999 to 2011 forms the core of the ETH Zurich/FOAG collection (personal communication Zeller). The collection was complemented with ten commercially available accessions, germplasm sourced from existing public collections at academic institutions, including the Bavarian State Research Center for Agriculture (8 accessions), Crop Research Institute Drnovská (4), University of Hohenheim (6), Laimburg Research Centre (3), the gene bank of IPK Gatersleben (12), ETH Zurich (16) and INRAE (1), as well as from non-governmental organizations, including ProSpecieRara (7), VERN (1) and GraanGeluk (1). Metadata describing the accessions are summarized in **Supplementary Table 1**. The accessions were collected from a wide geographical range, including North and South America, Europe, Asia and Oceania, with most accessions originating from Europe and East Asia. Some accessions can be associated with specific breeding programs, however, for most of the accessions, no information on breeding or selection history is available.

### 2.2 Seed multiplication

Seed multiplications were conducted from 2019 to 2023. Depending on the amount and quality of original seed, multiplication included propagation in isolation tents in the greenhouse, isolation tents in the field and isolated (>1 km distance to next common buckwheat cultivation) locations in home gardens (**Figure 1**). Greenhouse multiplications were used for accessions with low amounts of seed and/or seed with low germination rate. Accessions, for which fewer than five plants germinated, were not included in the multiplication nor the collection. Accessions with enough seed to grow 3 m^2^ (i.e., more than 15 g) were multiplied in the field.

**Figure 1:**
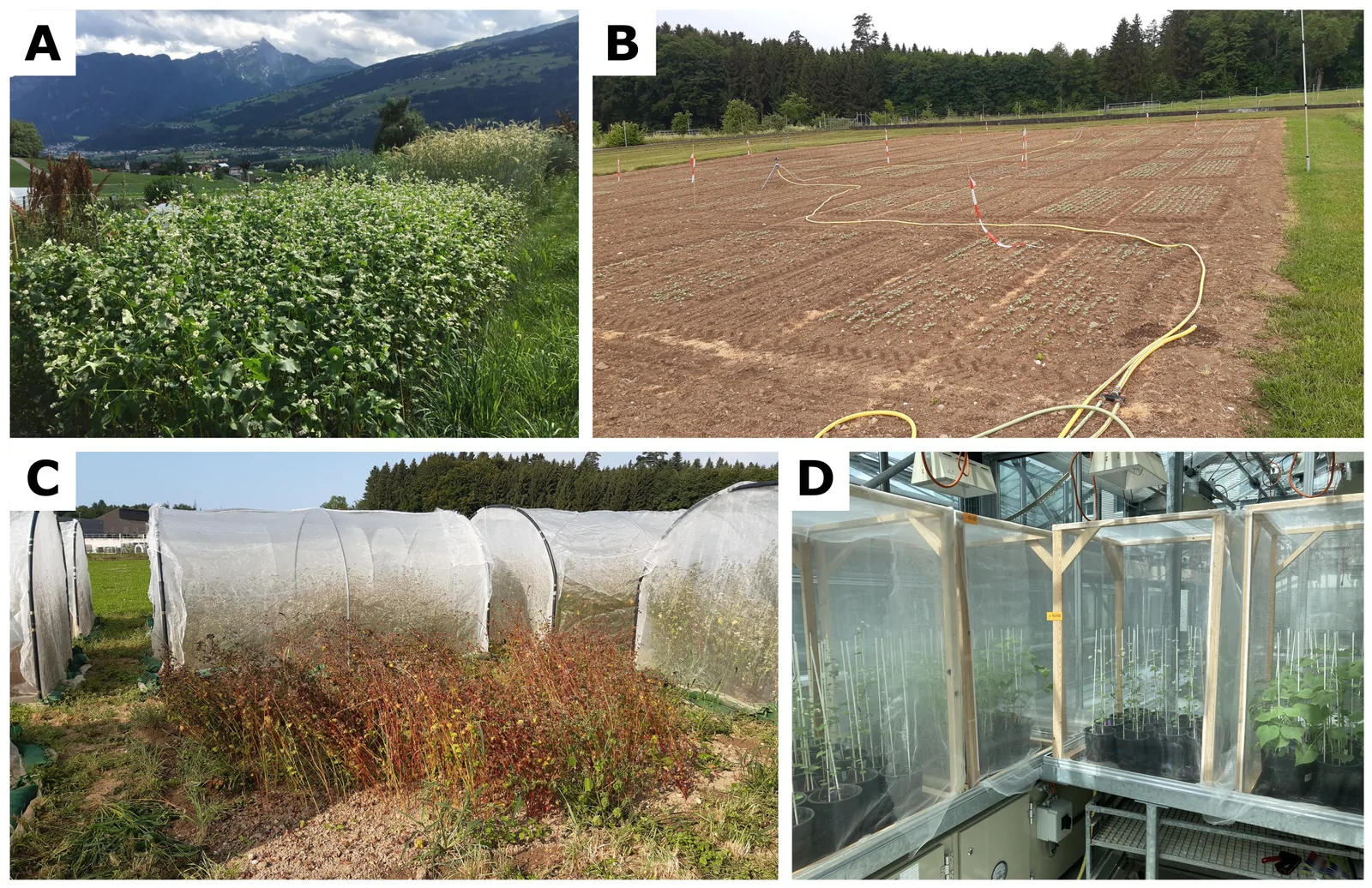
Pictures showing the different methods of common buckwheat multiplication used to propagate the ETH Zurich/FOAG collection. **(A)** Home garden multiplication in spatially isolated locations. **(B, C)** Field propagation in isolation tents. **(D)** Greenhouse multiplication in isolation tents. Photos: Stephan Stutz, Fabian Hess.

For greenhouse multiplication, seed were soaked in 2.5% sodium hypochlorite (Duso Javel, Roesch, St. Gallen, Switzerland), rinsed with water and germinated on wet tissue paper. The tissue paper was placed in a closed plastic container and kept at room temperature. Water was added when the paper started drying before germination occurred. After germination, plantlets were transplanted into 2.9 l plastic pots filled with a substrate mix of 25% sterilized soil, 25% bark compost, 20% washed sand and 30% white peat (premixed substrate available from Ricoter, Aarberg, Switzerland), with each pot containing two to three plants. To prevent cross-pollination between multiple accessions grown in the same greenhouse chamber, plants were separated using wooden frames covered with nets (mesh size 0.8 × 0.8 mm, Hortuna, Sursee, Switzerland) (**Figure 1D**). To ensure pollination between plants, *Calliphoridae* flies (Andy’s Fischershop, Zurich, Switzerland; Profiterr Schreiber, Widen, Switzerland) were added every one to two weeks during flowering. When > 80% of seed reached maturity, plants were cut and seed were separated from stems by hand and dried at 30 °C. The seed were cleaned using a laboratory thresher (type Allesdrescher K35, Baumann Saatzuchtbedarf, Waldenburg, Germany).

For multiplication in the field, seed were sown at a rate of 180 seed m^-2^ using a plot drill (type TC, Wintersteiger, Ried, Austria). To prevent cross-pollination, the plots were covered with mesh tents (mesh size 0.8 × 0.8 mm, Hortuna, Sursee, Switzerland) and *Calliphoridae* flies were added for pollination as in the greenhouse. Once plants within a tent were ripe (defined as >80% of seed having turned dark), tents were removed and plants harvested by hand. Harvested plants were processed the same way as plants from the greenhouse.

For multiplication in home gardens, a similar procedure as for the field multiplication was used. However, no tents or addition of flies was needed, as separation of accessions was achieved by spatial separation. To ensure that the same procedure was followed in all home gardens, a detailed protocol covering sowing, maintenance and harvest of the multiplication plots was prepared and shared with participants.

### 2.3 Sampling and sequencing

In 2023 and 2024, all 170 common buckwheat accessions were grown in the field at the Plant Research Station of ETH Zurich in Eschikon, Switzerland (47.449 N, 8.682 E, 520 m a.s.l.). In each year, 100 randomly selected plants per accession were sampled by collecting one young, lesion-free leaf per plant resulting in a total of 200 leaves per accession. Previous research performed in common buckwheat (Nay et al., 2020) and forages (Tamura et al., 2022; Nay et al., 2023) suggest that samples based on 100 to 200 leaves are appropriate for the calculation of GWAFFs. From each leaf, a 3-mm diameter disk was cut with a punch plier (BGS technic KG, Wermelskirchen, Germany), and the 100 disks per accession were pooled to form one composite sample per accession and year. In 2024, five accessions (FE003, FE090, FE175, FE213 and FE275) were selected as controls to quantify technical variation introduced at different stages of the genotyping workflow (i.e., during sampling, DNA extraction and sequencing). For each of these accessions, i) two technical replicates of the 2023 composite samples were re-sequenced in 2024 to investigate sampling effects on GWAFF results; ii) the 2024 composite sample was extracted and processed as a technical replicate to evaluate variation associated with DNA extraction across plates and/or well positions; and iii) after DNA extraction, DNA from one technical replicate was split by transferring half of the extract into a second well and sequenced independently to test for sequencing-related bias. Overall, this design yielded six samples per control accession.

The samples were stored in 1.2 ml 8-strip reaction tubes (Starlab, Affoltern am Albis, Switzerland) and freeze-dried before shipping to LGC (Teddington, UK) for DNA extraction, library construction and sequencing. The ddRAD libraries were prepared using the restriction enzymes *Pst*I and *Ape*KI, following LGC’s inhouse protocol (Arvidsson et al., 2016). Sequencing was performed on an Illumina NovaSeq 6000 (Illumina, San Diego, USA) with a target of 7 M paired-end reads (2×150 bp) per sample.

### 2.4 Genotyping

Sequencing reads were demultiplexed by LGC using bcl2fastq (v2.20) and delivered as FASTQ files. Adapter sequences were trimmed using HTStream (v1.3.0) and the processed reads were mapped to haplome 2 of the genome assembly of Tuka (Tuka_h2, Hess et al., 2026) using bowtie2 (v2.5.1, Langmead and Salzberg, 2012). Tuka_h2 represents an individual of accession FE213 (Devyatka), which is of European origin and thus assumed to represent the majority of the accessions in the collection. The resulting BAM files were sorted with samtools (v1.17, Danecek et al., 2021) and used for single nucleotide polymorphism (SNP) calling with the bcftools ‘mpileup’ and ‘call’ modules (v1.20, Danecek et al., 2021), adjusting the maximum read depth per site to 10,000. The resulting VCF file was used to extract allele frequencies (FORMAT/VAF) as well as allelic depths (FORMAT/AD). The allele frequencies were filtered using thresholds, which were defined based on the distribution and quality of the sequencing data (**Supplementary Figure 1**). Allele frequencies derived from allele depths below 30 per accession were set to missing, and SNPs with more than 5% missing data across accessions were filtered out. In addition, SNPs with a minor allele frequency below 5% or a standard deviation of allele frequencies below 0.05 were removed, as they were considered rare or invariant SNPs. This filtered dataset was used to assess the reproducibility of GWAFF results by comparing samples collected in 2023 and 2024. For further analyses, the sorted BAM files from the same accession across both years were merged. SNP calling and filtering were then repeated on the merged dataset following the same steps described above.

### 2.5 Phenotyping

In the same field experiment as the leaf samples for DNA extraction were collected, flower morphology (as a proxy of self-compatibility) and determinate growth habit of accessions were characterised by assessing their respective phenotypes as described in Matsui et al. (2003) and Fesenko et al. (2018), respectively. Moreover, seed of all accessions were harvested at the end of the growing season and classified according to their dominant color as black, brown or silver. The thousand grain weight (TGW) was assessed using a MARViN ProLine II seed analyzer (MARViTECH, Wittenburg, Germany).

### 2.6 Data analysis

Principal component analysis (PCA), construction of the phylogenetic tree as well as the generation of graphics were all performed in R (v4.4.0, R CORE TEAM, 2024). The phylogenetic tree was constructed following Gondro (2015). Genetic distances between accessions were estimated with the allele sharing method while hierarchical clustering of the accessions and the construction of phylogenetic trees were performed with the functions ‘hclust’ and ‘as.phlyo’ implemented in the R libraries ‘stats’ and ‘ape’, respectively. Population structure was analyzed using NGSadmix (Skotte et al., 2013) with the number of inferred clusters ranging from K = 2 to K = 10. NGSadmix requires the genotype input to be recoded as genotype likelihoods in Beagle format. Therefore, raw SNP data from the VCF files were filtered for bi-allelic SNPs (--min-alleles 2 --max-alleles 2), minimum minor allele frequency of 0.05 (--maf 0.05) and a minimum quality score of 30 (--minQ 30) using vcftools (v0.1.15, Danecek et al., 2011). Following the filtering, the VCF file was split by chromosomes, and the genotype likelihoods were extracted in Beagle format (--BEAGLE-PL) using VCFtools. The resulting likelihood files of each chromosome were concatenated into a single genome-wide file and used as input for NGSadmix for the calculation of cluster proportions of all accessions. Accessions with a probability of membership to a cluster of more than 50% were assigned to this cluster, while accessions that did not reach this threshold were classified as unassigned. To determine the number of clusters to include in downstream analyses, a ΔK analysis (Evanno et al., 2005) was performed. The selection of K was based on both the statistical evidence provided by this analysis and prior knowledge of the accessions, including their geographic origins and phenotypic characteristics. The diversity indices nucleotide diversity (π), Theta Watterson (θ) and Tajima’s d as well as fixation indexes (F_ST_) were calculated using grenedalf (Czech et al., 2024) based on allele frequencies calculated and filtered as described above. The “--sample-group-merge-table” option of grenedalf was used to calculate diversity indices for groups of accessions (such as clusters or breeding programs). Threshold values for F_st_ values representing highly differentiated SNPs were defined as 0.3% of SNPs with highest F_st_ values (i.e., > 0.6) for K = 2 and 0.015% for the analysis of K = 7 (i.e., F_st_ > 0.75). All genes annotated in the reference genome sequence Tuka within 20 kb around highly differentiated SNPs were extracted, including their functional annotation and the predicted protein sequence for further analysis.

## 3 Results

### 3.1 Seed multiplication

Over the five years of seed multiplication, a total of 114 multiplications were conducted in the greenhouse, 158 in the field and 15 in home gardens (**Supplementary Table 2**). The seed produced during these multiplication cycles, together with the original seed in the collection, was sufficient for a total of 170 accessions to be sown in the field and thus included in this study. The resulting collection of 170 accessions covers a wide range of geographic origins with a focus on Europe and Asia (**Supplementary Table 1**). Moreover, the accessions include nine self-compatible accessions and eleven accessions with determinate growth habit. Furthermore, for 81 of these accessions, seed of sufficient quantity and quality (i.e., with a germination rate above 90%) were produced to be stored in and made available through the Swiss National Gene Bank PGRFA (www.pgrel.admin.ch/pgrel).

### 3.2 Sequencing and SNP calling

The ddRAD sequencing produced on average 7.7 M and 7.0 M read pairs per sample in 2023 and 2024, respectively, with individual values ranging from 1.0 M to 17.7 M read pairs per sample. Mapping of reads merged across both years to Tuka resulted in an average mapping rate of 92% (**Supplementary Table 3**). The SNP calling produced an initial amount of 118,172 SNPs, which was reduced to 52,182 high quality SNPs after filtering. These SNPs were distributed over all eight chromosomes (**Supplementary Figure 2**) with chromosome-specific counts ranging from 5,815 SNPs on chromosome 7 to 7,923 SNPs on chromosome 6.

The PCA comparing GWAFF data between years showed no observable year effect in the first two PCs, which together explained 27% of the variance. A minor year effect was evident in the third PC; however, this component accounted for only 3% of the total variance (**Supplementary Figures 3A, B**). No year effects were detected in the PCA based on the control accessions alone (**Supplementary Figures 3C, D**). Analysis of pairwise genetic distances further revealed that samples collected in different years from the same accession were more closely related than samples from different accessions, except for two groups of genetically very similar accessions. One group comprised seed lots obtained from a German mill (FE281-FE286) together with the accessions FE235, FE224 and FE209, while a second group includes eight closely related accessions from central Europe (**Supplementary Figure 3E**). Overall, these analyses demonstrate high consistency of the genotyping results between years. Therefore, all subsequent analyses are based on the merged genotyping dataset combining reads from 2023 and 2024 for each accession.

### 3.3 Phylogenetic clusters

The ΔK analysis gave strong statistical support that K = 2 represents the appropriate number of clusters within the population (**Supplementary Figure 4**). However, as K = 3 and K = 7 provide additional insights into the population structure of the collection, these results were also included in the analysis and discussion. The population structure analysis with NGSadmix (K = 2) resulted in the definition of two main clusters. Most of the accessions show a strong identity to one of the two clusters with 81% of the accessions showing a group identity of 80% or higher (**Figure 2D**). The two clusters correspond well with the broad geographic origin of the accessions, with cluster 1 comprising accessions predominantly from Europe and cluster 2 consisting mainly of accessions from Asia (Fisher’s exact test: *p* < 0.001). Out of the nine self-compatible accessions in the collection, eight were assigned to cluster 2, while only accession FE204 was assigned to cluster 1. Increasing the number of clusters to three (K = 3) resulted in five self-compatible accessions formerly belonging to cluster 2 forming a subcluster.

**Figure 2:**
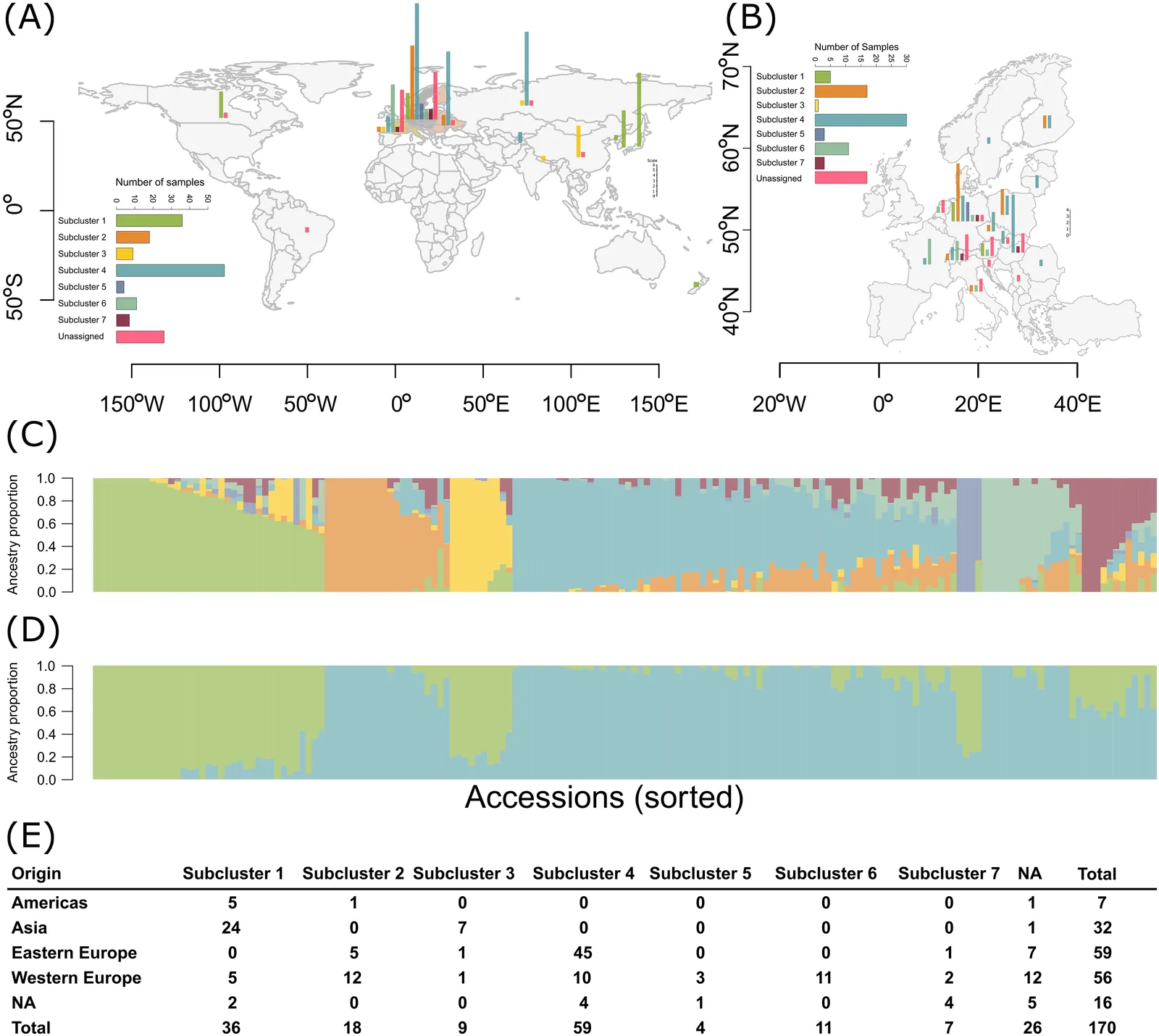
Clusters and phylogeography of the 170 common buckwheat accessions of the ETH Zurich/FOAG collection. **(A, B)** Origin of accessions in the collection. The height of bars indicates the number of accessions, while the colors refer to subclusters. **(A)** World-wide distribution of accessions with known origin. B) Distribution of accessions within Europe **(C, D)** Proportion of ancestral group identity for each accession calculated by NGSadmix with **(C)** K = 7 and **(D)** K = 2, ancestry proportions shown in **(C)** were used to sort accessions in both figures **(E)** Contingency table of accessions from different geographic origin belonging to subclusters identified by NGSadmix (K = 7). Unassigned accessions do not have a group identity of over 50% for any subcluster indicating mixed ancestral origin. The geographic distributions of the subclusters differ significantly from each other (Fisher’s exact test: *p* < 0.001).

When the number of clusters was set to seven (K = 7, hereafter subclusters), cluster 1 (“Europe”) differentiated into one subcluster dominated by Eastern European accessions (Subcluster 4) and two subclusters containing mainly accessions of Western European origin (Subclusters 2 and 6, **Figures 2A–C, E**). The two subclusters mainly originating from Western Europe could be differentiated by the color of the seed hull. Subcluster 6 contained accessions with silver-colored seed and Subcluster 2 consisted of accessions with brown and black seed (**Figure 3**).

**Figure 3:**
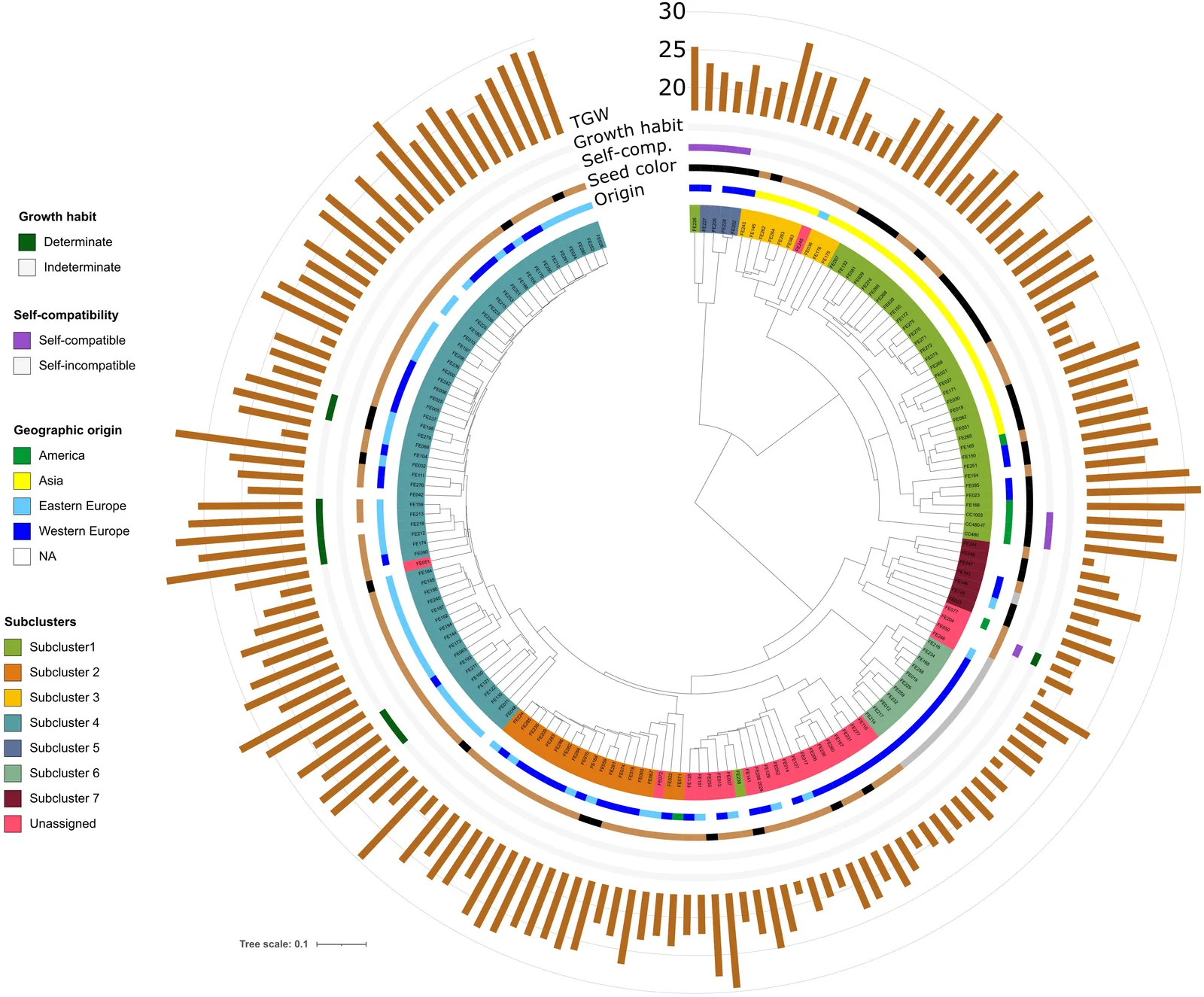
Phylogram of the 170 common buckwheat accessions of the ETH Zurich/FOAG collection. The accession names are colored according to their predicted subcluster based on NGSadmix (K = 7). Origin: The colors refer to the continents of origin of the accession: green = Americas, yellow = Asia, light blue = Eastern Europe, dark blue = Western Europe. Seed color: The seed colors black, brown and grey found in the ETH Zurich/FOAG collection are represented by their respective colors. Self-comp.: Self-compatible accessions are highlighted in violet. Growth habit: Accessions showing determinate growth habit are highlighted in dark green. TGW: The brown bars represent the thousand grain weight (g) of accessions.

Cluster 2 (“Asia”) split into two ancestral subclusters with the first one mainly consisting of accessions from Japan and Korea (Subcluster 1) and a second subcluster containing accessions from China and Nepal (Subcluster 3, **Figures 2A, E**). The remaining two subclusters contained five self-compatible accessions (Subcluster 5) and a group of accessions with diverse geographic backgrounds containing many accessions received from gene banks or botanical gardens (Subcluster 7).

The analysis of the predicted ancestral subgroups on country level revealed that accessions from countries in Asia and Eastern Europe can usually be traced back to a single ancestral (sub)cluster, while accessions from Western European countries usually relate to several different subclusters. **Figure 2B** shows the subcluster identities of accessions from Western Europe as well as the Western part of Eastern Europe, illustrating the diverse ancestral composition of accessions in this region. However, certain distribution patterns of accessions belonging to specific ancestral subgroups could be identified: While accessions of Subcluster 6 are limited to Western European countries (France, Switzerland, Italy and the Netherlands), accessions of Subclusters 2 mainly originate from Central Europe and Subcluster 4 consists largely of accessions from Eastern Europe. Accessions with diverse TGW are found in all ancestral groups, however, Subcluster 6 shows the lowest mean TGW of all subclusters (*t-*test, *p* < 0.001).

### 3.4 Diversity analysis

Nucleotide diversity (π) on the accession level indicated very large differences between accessions, ranging from 0.01 to 0.33. The lowest nucleotide diversity was found in the self-compatible accessions FE202, FE228, FE204, FE205, FE227 and FE226, with all these accessions having π values below 0.2. The remaining self-compatible accessions CC480, CC480-IT and CC1003 showed π values between 0.21 and 0.26, which is within the range of values observed in self-incompatible accessions. This pattern corresponded with the π values found in the seven subclusters, where all the subclusters showed π values within the range of 0.30 to 0.33, except for Subcluster 5, which showed a much lower value of 0.12 and contained exclusively self-compatible accessions (**Table 1**). The π values of accessions that are part of breeding programs (as defined in **Supplementary Table 1**) showed values around 0.31, except for the program of Kade Research Ltd. that relies on self-compatible accessions and shows a lower π value of 0.26 (**Table 1**). Accessions that were multiplied in the greenhouse showed a slightly but significantly (*t*-test, *p* < 0.001) lower average π value of 0.28 compared to accessions that were multiplied in the field or were not multiplied during the project (π = 0.29). Values for Theta Watterson fell within a relatively narrow range from 0.10 in Subcluster 4 to 0.14 in Subcluster 5, while Tajima’s D followed largely the pattern of nucleotide diversity with most subclusters and breeding programs showing values between 4.09 (breeding program National Agriculture and Food Research Organization NARO) and 6.01 (Subcluster 1) while groups including exclusively self-compatible accessions (Subcluster 5 and breeding program Kade Research Ltd.) having lower values of -0.42 and 3.07, respectively.

**Table 1:** Diversity indices of subclusters, known breeding programs and the entire ETH Zurich/FOAG germplasm collection of common buckwheat.

Population	Theta π	Theta Watterson	Tajima’s D
Subcluster			
Subcluster 1	0.33	0.11	6.00
Subcluster 2	0.31	0.11	4.99
Subcluster 3	0.33	0.12	4.83
Subcluster 4	0.32	0.10	6.01
Subcluster 5	0.12	0.14	-0.42
Subcluster 6	0.30	0.12	4.29
Subcluster 7	0.31	0.13	4.39
Unclassified	0.33	0.11	5.69
Breeding programs			
Kade Research Ltd.	0.26	0.13	3.07
Orel	0.31	0.12	4.33
NARO	0.31	0.13	4.09
Zacherl	0.31	0.12	4.44
ETH Zurich/FOAG collection			
Entire collection	0.34	0.12	5.25

### 3.5 Highly differentiated genomic regions

The F_ST_ analysis of the two main ancestral groups (NGSadmix, K = 2) revealed seven SNPs with values above 0.6, four of them being located within one hundred base pairs towards the end of chromosome 5. The remaining three SNPs were located on chromosomes 2 and 7 (**Supplementary Table 4**). All seven SNPs were also found in at least one pair-wise comparison between the seven ancestral subgroups (F_ST_ > 0.6). High F_ST_ values (>0.75) were mainly observed in the comparisons between subclusters from distant geographic origins, while pair-wise comparisons between ancestral groups with similar geographic origins (i.e., within Asia or Europe) did not show F_ST_ values above 0.75. The analysis showed that Subcluster 5 (which only contains self-compatible accessions) is very strongly differentiated from all other groups with many SNPs showing F_ST_ values above 0.9. Excluding comparisons involving Subcluster 5 revealed a total of 162 SNPs showing F_ST_ values above 0.75 in any of the pairwise comparisons. By merging SNPs in proximity (< 10 kb apart from each other) or showing up in more than one comparison, a total of 52 genomic regions were identified, in which SNPs with F_ST_ values above 0.75 were observed (**Supplementary Table 5**). Search for annotated genes within 20 kb around the identified SNPs in the reference genome Tuka revealed seven annotated genes around SNPs from the comparison of the two main clusters (**Supplementary Table 6**) and 71 from the comparison of the seven subclusters, including genes associated with flowering time and photoperiod such as *FRIGIDA* and genes linked to the regulation of auxin transport (PIN-likes) and Ca^2+^ signals (Glutamate receptor like, **Supplementary Table 7**).

## 4 Discussion

### 4.1 Phylogenetic clusters of common buckwheat: diverse origins in Western Europe

Our results contribute to elucidate the phylogeography of common buckwheat and reveal patterns of germplasm distribution and exchange. In particular, the data suggest that common buckwheat accessions in Europe and Asia are clearly differentiated with 90% of European accessions being related to main cluster 1, while all Asian accessions can be traced back to cluster 2. The observed population structure is likely the result of a combination of historical migration, founder effects and restricted gene flow among major cultivation regions.

Within Asia, analysis of subclusters indicates strong genetic differentiation between accessions from Northern Asia (Japan and Korea) and the Himalayan region (China and Nepal). The presumed center of origin of common buckwheat is located in Southern China (Ohnishi, 1995) from where it is reported to have spread to both North-eastern Asia and Europe many hundreds to thousands of years ago (Campbell, 1997; Verschuer, 2016). Only a subset of the available genetic diversity may have accompanied each migration event. Such founder effects could explain the formation of the distinct, geographically defined subclusters that were observed in our collection. Subsequent geographic isolation and limited exchange of germplasm over long distances could have resulted in further divergence between subclusters through genetic drift and local adaption. On the other hand, within regions such as Japan, Korea, the Himalayas as well as Eastern and Western Europe, exchange of germplasm appears to have been extensive resulting in comparatively low differentiation of accessions within these regions.

The strong differentiation between geographically and climatically distant regions is also observable in the analysis of the F_ST_ values (**Supplementary Table 5**). High F_ST_ values are mainly found when comparing subclusters from Europe with subclusters from Asia, while subclusters within the same continents show fewer SNPs with high differentiation. While the analysis is not able to differentiate whether genetic differences are caused by genetic drift or selection, inspection of the genome annotation around highly differentiated genomic regions revealed that some of these regions are closely linked to annotated genes with known functions influencing flowering time or flower development such as *FRIGIDA* (Johanson et al., 2000), which shows high differentiation between Subcluster 3 (Himalayan region) and Subcluster 6 (Western Europe). Similarly, genomic regions that are highly differentiated between Subclusters 1 (East Asia) and 7 (Central and Western Europe) harbor genes annotated as PIN-likes and glutamate receptor-likes, which have been described to be involved in plant growth and development (Sun et al., 2020) and response to environmental stimuli (Yu et al., 2022), respectively. These differentiations could therefore hint to an adaptation of the accessions to cropping systems in different climatic regions.

The phylogeographic patterns revealed in this study confirm the reliability of the genotyping approach applied. This is in line with findings of Nay et al. (2020), who showed that the genotyping of accessions consisting of genetically diverse individuals using ddRAD sequencing to calculate GWAFFs for common buckwheat is reliable. To add to the reliability of our genotyping data, leaf samples were collected in two different years and genotyped independently from each other. As the two sequencing rounds yielded comparable results, this further substantiates that the genotyping process is highly repeatable (**Supplementary Figures 3A, B**). As accessions consist of genetically diverse plants, different environmental conditions could lead to changing selection pressures between years. This could result in shifts of the genetic composition of the populations. While our data does not indicate such selection effects, their possibility should be considered if our genotyping data is used in different contexts. This could be especially relevant if seed are further multiplied and selection or genetic drift could affect the genetic composition of the accessions.

Interestingly, while accessions from geographically defined regions in Asia and Eastern Europe share a single gene pool, i.e., can be traced back to a single subcluster, this is not the case in Western Europe, hinting at differences in historic gene flow within these regions. Several subclusters coexist in most Western European countries (and some Eastern European countries such as Poland and the Czech Republic). Moreover, the high prevalence of accessions with no clear identity to one of the subclusters show that many Western European accessions are of mixed origin. This pattern is consistent with repeated introductions of germplasm through trade routes and farmer-mediated seed exchange, leading to continuous admixture among previously differentiated gene pools. The main subclusters present in Western Europe are Subclusters 2, 4 and 6, with Subcluster 4 sharing the same origin with accessions from Eastern Europe, while accessions belonging to Subcluster 2 are exclusively found in Central and Western Europe. In contrast, Subcluster 6 is not only associated with Western European origin but also with a specific seed phenotype; defined by silver colored seed with low thousand grain weight.

Combining data about these phylogeographic patterns with knowledge on common buckwheat utilization can shed light on how these distribution patterns might be rooted in history and the culinary preferences of different regions. To illustrate these patterns, Switzerland, a country with accessions tracing back to many different subclusters (**Supplementary Table 1**), can serve as an example. In Switzerland, accessions matching the phenotype associated with Subcluster 6 are traditionally referred to as “Scottish buckwheat”, while accessions with dark seed were called “Silesian buckwheat” (Schilperoord, 2017). These terms might be interpreted as referring to a Western or Eastern origin of said accessions, respectively. In some alpine regions black hull residues in the white buckwheat flour is considered a quality trait and might thus result in a preference for dark colored, “Silesian” accessions (Kreft et al., 2007) that are associated with subcluster 4. Moreover, “Silesian” buckwheat has been reported to be easier to dehull due to its seed shape (Schilperoord, 2017). This trait might be especially important in regions (such as Central and Eastern Europe), where common buckwheat is traditionally consumed as dehulled grains (Kreft et al., 2007). The “Scottish buckwheat” on the other hand is considered to have originated from France (Schilperoord, 2017), where it is cherished for this flour quality and light grey color (Costanzo, 2019; Morel, 2019). Therefore, regional differences in the use of common buckwheat across Europe might have contributed to the separation of subclusters with countries like Switzerland receiving influence from their Eastern and Western neighbors both in terms of genetic resources and culinary traditions.

### 4.2 Sources of diversity for common buckwheat breeding

The multiplication of true-to-type seed of cross-pollinating crops is a challenging but necessary step to preserve the qualities of individual accessions and to ensure that diverse germplasm remains available for breeding in the future (Weissmann et al., 2023). As common buckwheat is mainly pollinated by insects (Goodman et al., 2001; Jacquemart et al., 2007; Liu et al., 2020), it has been suggested that double layers of nets are required to protect outcrossing of different common buckwheat accessions multiplied in close proximity (Campbell, 1997). As we used only a single layer of net to prevent cross-pollination during field multiplication, potential cross contamination between accessions could be an issue. As we did not genotype accessions between multiplication cycles, it is not possible to quantitatively assess whether this occurred. However, our data suggest that genetic differences between accessions from different origins could be preserved during multiplication, thus indicating that pollination within accessions was by far the dominant mode of procreation. A further issue of true-to-type multiplication of cross-pollinating crops is a potential genetic bottleneck when germination rate is low, or seed stocks are limited. Accessions that were multiplicated in the greenhouse, which can be seen as a proxy for a low initial seed stock, was associated with a slight reduction of nucleotide diversity by 0.01. The observed reduction of nucleotide diversity in our accessions was very low and indicates that our initial seed stock was large enough to preserve most of the diversity of the respective accessions. However, these results indicate that lower initial seed stocks or repeated multiplication cycles based on small seed amounts could induce genetic drift and result in a loss of diversity (Fu, 2024). In this study, we therefore show a workable way to conserve and multiply large numbers of accessions of an outcrossing crop to make these genetic resources available for field research and agronomic utilization.

Our results suggest that there has been limited exchange of common buckwheat germplasm outside of geographically limited regions. This is also true for the accessions associated with breeding programs included in our collection. A particularly well documented breeding program exists at the All-Russia Research Institute of Legumes and Groat Crops in Orel (Fesenko et al., 2018). Several accessions that have been described to play important roles at different stages of this breeding program were included in the present study, including FE104 (named K-3466; representing a local Russian population), FE237 (Bogatyr; a variety released in Orel in 1938), FE144 (Kazanka, a KS (Krasnostreletskiy) type population from 1989) and FE090 (Ballada 2, a “limited secondary branching” population from 1985). As illustrated in **Figure 3**, these four accessions represent a large part of the genetic diversity found in Subcluster 4. Thus, most accessions found in Eastern Europe (including all accessions with determinate growth habit) appear to originate from germplasm associated with (or at least closely related to) Orel’s breeding program. While documentation is more limited in the case of the breeding program at the National Agriculture and Food Research Organization (NARO) in Japan, newer accessions released by NARO (FE266, FE267 and FE268) are closely related to traditional genetic resources from the Japan as described by Katsube-Tanaka (2016) such as FE020 (Miyazaki Ootsubu), FE274 (Shinshu-Oosoba) and FE273 (Kitawasesoba) that all belong to Subcluster 1. The self-compatible accessions bred by Kade Research Ltd. in Canada (CC480, CC408-IT and CC1003) also fall into Subcluster 1 and can therefore be assumed to trace back to Japanese and Korean accessions. This relationship is likely to stem from the use of widely used North American accessions (Parde et al., 2003; Pavek, 2016) such as FE166 (Manisoba) and FE165 (Manor). While these accessions are genetically closely related to accessions from China, Korea and Japan, the name ‘Manisoba’ might be an indication of their Japanese origin. While our collection does not provide a comprehensive overview of common buckwheat breeding programs, these examples indicate that the exchange of germplasm between phylogeographic regions could enrich local breeding programs. Inclusion of foreign genetic resources into breeding programs in Japan has been suggested to overcome limitations of the domestic genetic basis (Katsube-Tanaka, 2016). Several accessions bred in Southern Russia in Orel have achieved good agronomic performance in Western European climates (Strahm et al., 2019; Grimes et al., 2025), illustrating that the transfer of germplasm between regions can be successful.

The nucleotide diversities reported in this study were calculated based on polymorphic GWAFFs. Therefore, the absolute values are very high and not comparable to π values reported for plants in other studies (Ai et al., 2014). Nevertheless, comparisons between accessions, clusters, breeding programs as well as the entire collection within this study are valid and indicate that a high level of diversity is conserved even within individual accessions. This finding is also supported by other diversity indicators, as Theta Watterson is similar across clusters and breeding programs and Tajima’s D generally follows the same pattern as nucleotide diversity. However, the absolute values of Tajima’s D found in this study are exceptionally high with values up to 6.01. These values could be caused by reported statistical issues connected to their calculation based on allele frequency data and they thus should be interpreted with caution (Czech et al., 2024). Considering only self-incompatible accessions, it is notable that the overall nucleotide diversity found in the entire collection is only slightly higher compared to the two subclusters from Asia. Moreover, nucleotide diversity in breeding programs is similar compared to diversities found in subclusters. Therefore, our data indicates that breeding efforts in common buckwheat have not led to genetic bottlenecks that have extensively reduced genetic variation. This contrasts with many major self-pollinating crops, where intensive selection has narrowed the genetic base (Wouw et al., 2009) The predominantly outcrossing mating system of common buckwheat likely contributes to the maintenance of high within-population diversity. Moreover, this also conflicts with the idea that high diversity in outcrossing crops is usually an undesirable trait as it leads to inhomogeneities in the field. This is also illustrated by the observation that the accession with the highest nucleotide diversity in our collection (FE255) comes from a breeding project at University of Hohenheim in Germany. The success of this accession seems to be based more on the combination of the diversity of different accessions and less on the elimination of undesirable diversity by selection. A similar observation can be made by looking at nucleotide diversities of self-compatible accessions that are part of the breeding program at Kade Research Ltd. compared to other self-compatible accessions. The accessions bred for agronomic performance (CC480, CC480-IT, CC1003) all have higher nucleotide diversity compared to other self-compatible accessions. Based on our genotyping approach it is not possible to determine whether this high diversity stems from low homozygosity of individual plants or that accessions consist of a set of diverse highly homozygous lines.

### 4.3 From germplasm characterization to state-of-the-art breeding

Beyond describing global phylogeny and diversity patterns, the present study establishes a resource that can support modern breeding strategies for common buckwheat. Access to the ETH Zurich/FOAG collection through the Swiss National Gene Bank in combination with the GWAFFs provides opportunities to (i) link the genotypic information with multi-environment phenotypic assessments to enable the identification of genomic regions controlling agronomically relevant traits using genome-wide association studies, (ii) establish training populations for genomic prediction and selection, (iii) facilitate the establishment of a core collection of common buckwheat by providing a rational basis for selecting a representative subset of accessions that captures a maximum of genetic diversity while reducing efforts required for maintenance and (iv) create a foundation for pangenome studies by identifying distinct phylogeographic gene pools that need to be incorporated in order to appropriately represent species-wide diversity. Therefore, the genetic characterization presented here transforms the ETH Zurich/FOAG common buckwheat collection from a conservation resource into a valuable breeding resource that can contribute to increasing agricultural diversity.

## Data Availability

The ddRAD sequences aligned to the refrence genome assembly Tuka h_2 are deposited as BAM files in NCBI's Sequence Read Archive (SRA) at https://www.ncbi.nlm.nih.gov/sra/PRJNA1517880. The GWAFF matrix is available as a tab-separated values file from the ETH Research Collection at https://doi.org/10.3929/ethz-c-000805751.

## References

[B1] Agarwal B. (1981). Agricultural mechanisation and labour use: A disaggregated approach. Int. Labour Rev. 120, 115–127. Available online at: https://labordoc.ilo.org/permalink/41ILO_INST/mnptf4/alma995274634802676 (Accessed September 3, 2026).

[B2] Ai B. Kang M. Huang H . (2014). Assessment of Genetic Diversity in Seed Plants Based on a Uniform π Criterion. Molecules, 19 (12), 20113–20127. doi: 10.3390/molecules191220113 PMC6271616 25470277

[B3] Aii J. Nagano M. Penner A. G. Campbell G. C. Adachi T. (1998). Identification of RAPD markers linked to the homostylar (Ho) gene in buckwheat. Japanese J. Breed. 48, 59–62. doi: 10.1270/jsbbs1951.48.59

[B4] Arvidsson S. Fartmann B. Winkler S. Zimmermann W. (2016). Efficient high-throughput SNP discovery and genotyping using normalised genotyping-by-sequencing (NGBS). Available online at: https://biosearch-cdn.azureedge.net/assetsv6/efficient-high-throughput-snp-discovery-genotyping-ngbs-app-note.pdf (Accessed September 3, 2026).

[B5] Aufhammer W. Lee J. H. Kübler E. Kuhn M. Wagner S. (1995). Anbau und Nutzung der Pseudocerealien Buchweizen (*Fagopyrum esculentum* Moench), Reismelde (*Chenopodium quinoa* Willd.) und Amarant (Amaranthus) als Körnerfruchtarten. Die Bodenkultur - J. For. Land Management Food. Environ. 46, 125–140.

[B6] Byrne S. Czaban A. Studer B. Panitz F. Bendixen C. Asp T. (2013). Genome wide allele frequency fingerprints (GWAFFs) of populations via genotyping by sequencing. PloS One 8, 10. doi: 10.1371/journal.pone.0057438 23469194 PMC3587605

[B7] Campbell C. G. (1997). Buckwheat. Fagopyrum esculentum moench Promoting the Conservation and Use of Unterutilized and Neglected Crops (Rome, Italy: Institute of Plant Genetics and Crop Plant Research, Gantersleben/International Plant Genetic Resources Institue).

[B8] Costanzo A. (2019). DIVERSIFOOD-inventory of underutilised crops. Available online at: https://diversifood.eu/wp-content/uploads/2017/11/DIVERSIFOOD-WP21_Inventory-of-underutilised-crops.pdf (Accessed September 3, 2026).

[B9] Czech L. Spence J. P. Expósito-Alonso M. (2024). Grenedalf: Population genetic statistics for the next generation of pool sequencing. Bioinformatics 40, 5. doi: 10.1093/bioinformatics/btae508 39185959 PMC11357794

[B10] Danecek P. Auton A. Abecasis G. Albers C. A. Banks E. DePristo M. A. . (2011). The variant call format and VCFtools. Bioinformatics 27, 2156–2158. doi: 10.1093/bioinformatics/btr330 21653522 PMC3137218

[B11] Danecek P. Bonfield J. K. Liddle J. Marshall J. Ohan V. Pollard M. O. . (2021). Twelve years of SAMtools and BCFtools. GigaScience 10, 1–4. doi: 10.1093/gigascience/giab008 33590861 PMC7931819

[B12] Evanno E. Regnaut S. Goudet J. (2005). Detecting the number of clusters of individuals using the software STRUCTURE: A simulation study. Mol. Ecol. 14, 2611–26120. doi: 10.1111/j.1365-294X.2005.02553.x 15969739

[B13] FAO (2019). The state of the world’s biodiversity for food and agriculture. Eds. Bélanger J. Pilling D. ( Rome: FAO Commission on Genetic Resources For. Food. and Agriculture Assessments). doi: 10.4060/CA3129EN

[B14] Farooq S. Ul Rehman R. Pirzadah T. B. Malik B. Ahmad Dar F. Tahir I. (2016). Cultivation, agronomic practices, and growth performance of buckwheat. Mol. Breed. Nutr. Aspects. Buckwheat. 299–313. doi: 10.1016/B978-0-12-803692-1.00023-742574925

[B15] Fesenko A. N. Fesenko N. N. Romanova O. I. Fesenko I. N. (2018). “ Main morphological types of cultivated buckwheat populations in Russia,” in Buckwheat Germplasm in the World (London: Academic press), 235–248.

[B16] Fu Y. B. (2024). Will a plant germplasm accession conserved in a genebank change genetically over time? Front. Plant Sci. 15. doi: 10.3389/fpls.2024.1437541 39430894 PMC11487523

[B17] Gondro C. (2015). Primer to Analysis of Genomic Data Using R. Eds. Gentleman R. Hornik K. Parmigiani G. (Cham, Heidelberg: Springer International Publishing Switzerland). doi: 10.1007/978-3-319-14475-7

[B18] Goodman R. Hepworth G. Kaczynski P. McKee B. Clarke S. Bluett C. (2001). Honeybee pollination of buckwheat (*Fagopyrum esculentum* Moench) cv. Manor. Aust. J. Exp. Agric. 41, 1217–1221. doi: 10.1071/EA9900838477348

[B19] Grimes S. J. Afzal M. Tako R. Hahn V. Graeff-Hönninger S. Longin C. F. H. (2025). Buckwheat in Germany: The effect of variety and sowing date on agronomic traits. Agron. J. 117, 1–12. doi: 10.1002/agj2.7019242587375

[B20] Halbrecq B. Romedenne P. Ledent J. F. (2005). Evolution of flowering, ripening and seed set in buckwheat (*Fagopyrum esculentum* Moench): Quantitative analysis. Eur. J. Agron. 23, 209–224. doi: 10.1016/j.eja.2004.11.00642574925

[B21] Hess F. Chen Y. López M.-E. Colliquet A. Stoffel-Studer I. Mac V. . (2026). Near-complete, haplotype-resolved genome assembly of common buckwheat (*Fagopyrum esculentum* Moench). Sci. Data, 2026.03.30.715208. doi: 10.1038/s41597-026-08092-437880705

[B22] Hunt H. V. Shang X. Jones M. K. (2018). Buckwheat: A crop from outside the major Chinese domestication centres? A review of the archaeobotanical, palynological and genetic evidence. Veg. Hist. Archaeobot. 27, 493–506. doi: 10.1007/s00334-017-0649-4 31258253 PMC6560938

[B23] Hunter D. Borelli T. Beltrame D. M. O. Oliveira C. N. S. Coradin L. Wasike V. W. . (2019). The potential of neglected and underutilized species for improving diets and nutrition. Planta 250, 709–729. doi: 10.1007/s00425-019-03169-4 31025196

[B24] IPBES (2019). IPBES 2019. Summary for policymakers of the global assessment report on biodiversity and ecosystem services of the Intergovernmental Science-Policy Platform on Biodiversity and Ecosystem Services. IPBES secretariat, Bonn, Germany 56. doi: 10.5281/zenodo.3553579

[B25] Jacquemart A.-L. Cawoy V. Kinet J.-M. Ledent J.-F. Quinet M. (2012). Is buckwheat (*Fagopyrum esculentum* Moench) still a valuable crop today. Eur. J. Plant Sci. Biotechnol. 6, 1–10.

[B26] Jacquemart A. L. Gillet C. Cawoy V. (2007). Floral visitors and the importance of honey bee on buckwheat (*Fagopyrum esculentum* Moench) in Central Belgium. J. Hortic. Sci. Biotechnol. 82, 104–108. doi: 10.1080/14620316.2007.1151220537339054

[B27] Jha R. Zhang K. He Y. Mendler-Drienyovszki N. Magyar-Tábori K. Quinet M. . (2024). Global nutritional challenges and opportunities: Buckwheat, a potential bridge between nutrient deficiency and food security. Trends Food Sci. Technol. 145, 18. doi: 10.1016/j.tifs.2024.10436542574925

[B28] Johanson U. West J. Lister C. Michaels S. Amasino R. Dean C. (2000). Molecular analysis of FRIGIDA, a major determinant of natural variation in *Arabidopsis* flowering time. Science 290, 344–347. doi: 10.1126/science.290.5490.344 11030654

[B29] Joshi D. C. Chaudhari G. V. Sood S. Kant L. Pattanayak A. Zhang K. . (2019). Revisiting the versatile buckwheat: Reinvigorating genetic gains through integrated breeding and genomics approach. Planta 250, 783–801. doi: 10.1007/s00425-018-03080-4 30623242

[B30] Joshi D. C. Meena R. P. Chandora R. (2021). “ Genetic resources: Collection, characterization, conservation, and documentation,” in Millets and Pseudo Cereals (Duxford: Woodhead Publishing), 19–31. doi: 10.1016/B978-0-12-820089-6.00003-3

[B31] Katsube-Tanaka T. (2016). “ Buckwheat production, consumption, and genetic resources in Japan,” in Molecular Breeding and Nutritional Aspects of Buckwheat (London: Academic Press), 61–76. doi: 10.1016/B978-0-12-803692-1.00005-5

[B32] Klerk P. Couwenberg J. Joosten H. (2015). Pollen and macrofossils attributable to *Fagopyrum* in Western Eurasia prior to the late Medieval: An intercontinental mystery. Palaeogeogr. Palaeoclimatol. Palaeoecol. 440, 1–21. doi: 10.1016/j.palaeo.2015.08.02442574925

[B33] Kreft I. Ries C. Zewen C. (2007). Das Buchweizen Buch. 2nd edition (Leudelange: Islek ohne Grenzen EWIV).

[B34] Kreft I. Wieslander G. Vombergar B. (2016). “ Bioactive flavonoids in buckwheat grain and green parts,” in Molecular Breeding and Nutritional Aspects of Buckwheat, 161–167.

[B35] Langmead B. Salzberg S. L. (2012). Fast gapped-read alignment with Bowtie 2. Nat. Methods 9, 357–359. doi: 10.1038/nmeth.1923 22388286 PMC3322381

[B36] Li X. Siddique K. H. M. (2018). Future smart food - Rediscovering hidden treasures of neglected and underutilized species for zero hunger in Asia (Bangkok: FAO (Food and Agriculture Organization of the United Nations)).

[B37] Lichtenhahn M. Dierauer H. (2000). Buchweizen (Frick: Merkblatt Frick). Available online at: https://www.fibl.org/fileadmin/documents/shop/1163-buchweizen.pdf (Accessed September 3, 2026).

[B38] Liu C. Plaza-Bonilla D. Coulter J. A. Kutcher H. R. Beckie H. J. Wang L. . (2022). Diversifying crop rotations enhances agroecosystem services and resilience. Adv. Agron. 173, 1–7. doi: 10.1016/bs.agron.2022.02.00742574925

[B39] Liu R. Chen D. Luo S. Xu S. Xu H. Shi X. . (2020). Quantifying pollination efficiency of flower-visiting insects and its application in estimating pollination services for common buckwheat. Agric. Ecosyst. Environ. 301, 107011. doi: 10.1016/j.agee.2020.10701142574925

[B40] Mascher M. Jayakodi M. Shim H. Stein N. (2024). Promises and challenges of crop translational genomics. Nature 636, 585–593. doi: 10.1038/s41586-024-07713-5 39313530 PMC7616746

[B41] Mascher M. Schreiber M. Scholz U. Graner A. Reif J. C. Stein N. (2019). Genebank genomics bridges the gap between the conservation of crop diversity and plant breeding. Nat. Genet. 51, 1076–1081. doi: 10.1038/s41588-019-0443-6 31253974

[B42] Matsui K. Tetsuka T. Nishio T. Hara T. (2003). Heteromorphic incompatibility retained in self-compatible plants produced by a cross between common and wild buckwheat. New Phytol. 159, 701–708. doi: 10.1046/j.1469-8137.2003.00840.x 33873587

[B43] Miedaner T. Longin F. (2017). Unterschätzte Getreidearten. Einkorn, Emmer, Dinkel & Co (Clenze: Erling Verlag Agrimedia).

[B44] Morel K. (2019). Small-scale dehulling of buckwheat report of farm visits in Western France (March 2019) report. Louvain-la-Neuve: Earth and Life Institute.

[B45] Nay M. M. Byrne S. L. Pérez E. A. Walter A. Studer B. (2020). Genetic characterization of buckwheat accessions through genome-wide allele frequency fingerprints / Genetska karakterizacija vzorcev ajde z odtisi frekvence alelov v genomu. Folia Biologica Geologica 61, 17–23. doi: 10.3986/fbg0063

[B46] Nay M. M. Grieder C. Frey L. A. Amdahl H. Radovic J. Jaluvka L. . (2023). Multi-location trials and population-based genotyping reveal high diversity and adaptation to breeding environments in a large collection of red clover. Front Plant Sci. 14, 1128823. doi: 10.3389/fpls.2023.1128823 PMC10014843 36938037

[B47] Ohnishi O. (1995). “ Discovery of new *Fagopyrum* species and its implication for the studies of evolution of *Fagopyrum* and of the origin of cultivated buckwheat”, in: Proc. 6 Intl. Symp. Buckwheat, Japan, 1995, Ina: Shinshu University Press. 175–190.

[B48] Parde S. R. Johal A. Jayas D. S. White N. D. (2003). Physical properties of buckwheat cultivars. Can. Biosyst. Eng. / Le Genie Des. Biosyst. Au Canada 45, 6–9.

[B49] Pavek P. L. S. (2016). Plant guide for buckwheat (*Fagopyrum esculentum*)(Pullman, WA: USDA (United States Department of Agriculture)). Available online at: https://plants.usda.gov/DocumentLibrary/plantguide/pdf/pg_faes2.pdf.

[B50] Pingali P. (2007). “ Chapter 54 Agricultural mechanization: Adoption patterns and economic impact,” in Handbook of Agricultural Economics, Amsterdam NL, Oxford: North Holland. vol. 3. , 2779–2805. doi: 10.1016/S1574-0072(06)03054-4

[B51] R Core Team (2024). R: A language and environment for statistical computing (Vienna, Austria: R Foundation for Statistical Computing).

[B52] Salgotra R. K. Chauhan B. S. (2023). Genetic diversity, conservation, and utilization of plant genetic resources. Genes 14, 20. doi: 10.3390/genes14010174 36672915 PMC9859222

[B53] Schilperoord P. (2017). Kulturpflanzen in der Schweiz - Buchweizen (Alvaneu: Verein für alpine Kulturpflanzen). doi: 10.22014/97839524176-e7

[B54] Shah K. K. Modi B. Pandey H. P. Subedi A. Aryal G. Pandey M. . (2021). Diversified crop rotation: An approach for sustainable agriculture production. Adv. Agric. 2021 (1), 1–9. doi: 10.1155/2021/8924087

[B55] Skotte L. Korneliussen T. S. Albrechtsen A. (2013). Estimating individual admixture proportions from next generation sequencing data. Genetics 195, 693–702. doi: 10.1534/genetics.113.154138 24026093 PMC3813857

[B56] Škrabanja V. Kreft I. (2016). “ Nutritional value of buckwheat proteins and starch,” in Molecular Breeding and Nutritional Aspects of Buckwheat (London: Elsevier), 169–176.

[B57] Strahm S. Füglistaller D. Lädrach C. Enggist A. Thuet A. Luginbühl C. (2019). Buchweizenanbau in der Schweiz: Neue sorten für eine alte nischenkultur. Agrarforsch. Schweiz 10, 198–205.

[B58] Sun L. Feraru E. Feraru M. I. Waidmann S. Wang W. Passaia G. . (2020). PIN-LIKES coordinate brassinosteroid signaling with nuclear auxin input in *Arabidopsis thaliana*. Curr. Biol. 30, 1579–1588.e6. doi: 10.1016/j.cub.2020.02.002 32169207 PMC7198975

[B59] Tamura K. Arakawa A. Kiyoshi T. Yonemaru J. (2022). Genetic diversity and structure of diploid Italian ryegrass (*Lolium multiflorum* Lam.) cultivars and breeding materials in Japan based on genome-wide allele frequency. Grassland Sci. 68, 263–276. doi: 10.1111/grs.1236340046247

[B60] Verschuer C. (2016). Rice, Agriculture, and the Food Supply in Premodern Japan. Abington: Routledge. doi: 10.4324/9781315715605

[B61] Weissmann E. A. Yadav R. N. Seth R. Bhaskar K. U. (2023). “ Principles of variety maintenance for quality seed production,” in Seed Science and Technology: Biology, Production, Quality, Springer. 153–172. doi: 10.1007/978-981-19-5888-5_8

[B62] Wouw M. V. D. Kik C. Van Hintum T. Van Treuren R. Visser B. (2009). Genetic erosion in crops: Concept, research results and challenges. Plant Genet. Resources: Characterisation Utilisation 8, 1–15. doi: 10.1017/S147926210999006241292463

[B63] Yabe S. Iwata H. (2020). Genomics-assisted breeding in minor and pseudo-cereals. Breed. Sci. 70, 19–31. doi: 10.1270/jsbbs.19100 32351301 PMC7180141

[B64] Yang X. Xiong J. Du T. Ju X. Gan Y. Li S. . (2024). Diversifying crop rotation increases food production, reduces net greenhouse gas emissions and improves soil health. Nat. Commun. 15, 14. doi: 10.1038/s41467-023-44464-9 38172570 PMC10764956

[B65] Yu B. Liu N. Tang S. Qin T. Huang J. (2022). Roles of Glutamate Receptor-Like Channels (GLRs) in Plant Growth and Response to Environmental Stimuli. Plants 11, 1–16. doi: 10.3390/plants11243450 36559561 PMC9782139

[B66] Zeller F. J. Hsam S. L. K (2004). Buchweizen — die vergessene Kulturpflanze: Funktionelles Lebensmittel. Biologie in unserer Zeit. 34, 24–31. doi: 10.1002/biuz.20041024142587375

